# Expression levels and diagnostic value of serum GDNF, CEA and CA199 in patients with colorectal carcinoma

**DOI:** 10.5937/jomb0-44745

**Published:** 2024-04-23

**Authors:** Wang Jue, Liu Lulu, Zheng Yan, Sai Gu

**Affiliations:** 1 The First Affiliated Hospital of Chongqing Medical University, Department of Gastroenterology, Chongqing, China

**Keywords:** GDNF, CEA, CA199, colorectal carcinoma (CRC), diagnostic value, GDNF, CEA, CA199, kolorektalni karcinom (CRC), dijagnostička vrednost

## Abstract

**Background:**

To investigate the expression levels and diagnostic value of glial cell line-derived neurotrophic factor (GDNF), carcinoembryonic antigen (CEA) and carbohydrate antigen199 (CA199) in patients with colorectal carcinoma (CRC).

**Methods:**

50 CRC patients at our hospital from Feb. 2020 to Feb. 2021 were chosen as the malignant group, another 50 patients with benign colonic diseases were chosen as the benign group, and 50 healthy people who came to our hospital for physical examination during the same period were considered as the control group. Fasting peripheral venous blood was taken from all research subjects in the morning and tested by a fully-automated electrochemiluminometer to determine the GDNF, CEA and CA199 levels. The sensitivity and specificity of the combined detection of the three indexes for CRC were analyzed, and the receiver operating characteristic (ROC) curve was plotted to record the area under the curve (AUC).

**Results:**

The malignant group had remarkably higher CEA and CA199 levels (P<0.001) and a lower GDNF level (P<0.001) when compared with the benign and control groups. The sensitivity, specificity, positive predictive value and negative predictive value of the combined detection were 96.0%, 94.0%, 88.9% and 97.9%, respectively. Under combined detection, AUC (95% CI) = 0.950 (0.909-0.991), standard error = 0.021, and P<0.001.

**Conclusions:**

The combined diagnosis of serum GDNF, CEA and CA199 is a reliable method to improve the diagnostic accuracy of CRC, and this strategy can effectively reduce the missed diagnosis rate and has high application value in clinic.

## Abstract (sr)

**Uvod:**

Cilj je bio da se istraže nivoi ekspresije i dijagnostička vrednost neurotrofnog faktora (GDNF), karcinoembrionalnog antigena (CEA) i antigena ugljenih hidrata 199 (CA199) kod pacijenata sa kolorektalnim karcinomom (CRC).

**Metode:**

Za malignu grupu izabrano je 50 pacijenata sa CRC u našoj bolnici od februara 2020. do februara 2021. godine, za benignu grupu je izabrano još 50 pacijenata sa benignim oboljenjima debelog creva, a 50 zdravih ljudi koji su došli u našu bolnicu na fizički pregled tokom istog period su smatrani kontrolnom grupom. Periferna venska krv natašte je uzeta od svih ispitanika ujutru i testirana potpuno automatizovanim elektrohemiluminometrom da bi se odredili nivoi GDNF, CEA i CA199. Analizirana je osetljivost i specifičnost kombinovane detekcije tri indeksa za CRC, a kriva radne karakteristike prijemnika (ROC) je ucrtana da bi se zabeležila površina ispod krive (AUC).

**Rezultati:**

Maligna grupa je imala značajno više nivoe CEA i CA199 (P<0,001) i niži nivo GDNF (P<0,001) u poređenju sa benignom i kontrolnom grupom. Osetljivost, specifičnost, pozitivna prediktivna vrednost i negativna prediktivna vrednost kombinovane detekcije iznosile su 96,0%, 94,0%, 88,9% i 97,9%, respektivno. Pod kombinovanom detekcijom, AUC (95% CI) = 0,950 (0,909-0,991), standardna greška = 0,021 i P<0,001.

**Zaključak:**

Kombinovana dijagnoza serumskog GDNF, CEA i CA199 je pouzdana metoda za poboljšanje dijagnostičke tačnosti CRC, a ova strategija može efikasno smanjiti stopu promašene dijagnoze i ima visoku primenu u klinici.

## Introduction

Colorectal carcinoma (CRC), a malignant tumor originating from colorectal glandular epithelial cells, currently ranks 3rd in the incidence and 2nd in the mortality of malignant tumors worldwide, with more than 1 million new cases each year. Its incidence increases significantly with age, and about 30% of patients are diagnosed at an advanced stage, with clinical manifestations such as intestinal irritation, hematochezia, abdominal masses, intestinal obstruction, anemia, emaciation, and fatigue, and approximately 880,000 deaths each year [1] [2]. Improving the early detection rate is the key to increasing the 5year survival rate of patients. The detection of serum tumor markers has apparent advantages such as convenient and noninvasive examination, which can be used for the adjuvant diagnosis of CRC and provide a more comprehensive basis for the clinical selection of treatment plans [3]. Carcinoembryonic antigen (CEA) and carbohydrate antigen199 (CA199) are the most common serum tumor markers in the diagnosis of CRC. However, single detection of CEA or CA199 has a low positive rate and insufficient sensitivity and cannot obtain positive results [4], and their combined detection is needed. Glial cell line-derived neurotrophic factor (GDNF) is a substance secreted by enteric glial cells to promote the growth and differentiation of enteric neurons and maintain the homeostasis of the intestinal environment [5]. It can bind to the glial cell-derived neurotrophic factor receptor-α (GFRα) and RET receptor tyrosine kinase respectively to form the GDNF-GFRa-RET signaling pathway, which subsequently affects the occurrence and development of tumors [6]. In recent years, it has been reported that GDNF is closely related to the occurrence and development of digestive system tumors such as CRC and pancreatic cancer [7]. Meir et al. [8] have reported that GDNF has an abnormal methylation status in CRC patients, i.e., there is a prominent low expression of this gene, thereby differentiating colorectal cancer patients from healthy people. The combination of GDNF, CEA, and CA199 may improve the drawbacks of existing serum tumor markers and provide a reliable basis for pathological diagnosis by obtaining accurate results through a single blood test. Based on this, this study chose 50 CRC patients in order to investigate the expression levels and diagnostic value of serum GDNF, CEA, and CA199 in CRC patients.

## Materials and methods

### Research design

This retrospective study was conducted at our hospital between Feb. 2020 and Feb. 2021 to explore the clinical value of the combined detection of serum GDNF, CEA, and CA199 levels in the diagnosis of CRC. The study complied with the Declaration of Helsinki (2013) [9], and the patients were informed of the research purpose, significance, content, and confidentiality, and signed the informed consent.

### General data

The 50 CRC patients who were admitted to our hospital between Feb. 2020 and Feb. 2021 and met the diagnostic criteria of CRC in the Guidelines of Diagnosis and Treatment for Common Cancers in China [10] were chosen as the malignant group, undergoing full treatment, with complete clinical data. Patients with hearing impairment, speech impairment, unclear consciousness and other serious organic diseases were excluded.

### Methods and observation criteria

Fasting peripheral venous blood was taken from all research objects in the morning and the blood sample was centrifuged at 3000 r/min for 10 min to obtain the supernatant for detection. The MAGLUMI 4000 Plus fully-automated electrochemiluminometer (manufacturer: Xiamen Haifei Biotech Co., Ltd.; Guangdong Medical Products Administration Certified No.: 20132400466; origin: China; original matching reagents) was used to determine the GDNF, CEA and CA199 levels. The normal range was given in the kit as GDNF>45 ng/mL, CEA<3.4 ng/mL, and CA199<27 U/mL, and any index not within the normal range in the combined detection was considered a positive result. Based on the diagnosis results, the sensitivity and specificity of the combined detection of GDNF, CEA and CA199 for CRC were calculated, and the receiver operating characteristic (ROC) curve was plotted to record the area under the curve (AUC).

### Statistical analysis

In this study, the SPSS 26.0 statistical software (Armonk, State of New York, USA) produced by IBM corporation was used for data analysis, and ROC curve was adopted for analysis of diagnostic value. The research data included count data and measurement data, tested by χ^2^ and t test. The differences were considered statistically significant when P<0.05.

## Results

### Comparison of baseline data of all research objects

There was no statistical difference in gender, age and weight of research objects among three groups (P>0.05), and each item of baseline data was shown in Table 1.

**Table 1:** Comparison of baseline data of research subjects (n, x̄±s).

Indicators	Malignant group (n=50)	Benign group (n=50)	Control group (n=50)	P
Gender				
Male/female	35/15	32/18	34/16	>0.05
Age (years old)	65.30±5.19	65.58±5.46	65.72±5.41	>0.05
Weight (kg)	62.58±3.47	62.72±3.40	62.65±3.47	>0.05
Disease types				
Colon carcinoma	26 (52.00)	/		/
Cancer of rectum	24 (48.00)	/		/
Adenomatous polyp	/	20 (40.00)		/
Hyperplastic polyp	/	19 (38.00)		/
Inflammatory bowel disease	/	11 (22.00)		/
Dukes staging				
Stage A	5 (10.00)	/		/
Stage B	15 (30.00)	/		/
Stage C	16 (32.00)	/		/
Stage D	14 (28.00)	/		/
Bone metastasis	4 (8.00)	/		/
Lung metastasis	8 (16.00)	/		/
Liver metastasis	25 (50.00)	/		/
Extensive abdominal metastasis	8 (16.00)	/		/

### Comparison of GDNF, CEA, and CA199 levels

The malignant group had remarkably higher CEA and CA199 levels (P<0.001) and a lower GDNF level (P<0.001) compared with the benign and control groups, see Table 2.

**Table 2:** Comparison of GDNF, CEA, and CA199 levels (x̄±s). Notes: * P<0.001, compared to the benign group; ** P<0.001, compared to the control group.

Groups	n	GDNF (ng/mL)	CEA (ng/mL)	CA199 (U/mL)
Malignant group	50	30.35±17.82* **	24.97±17.31* **	47.79±18.24* **
Benign group	50	58.89±15.70**	3.00±0.75**	19.33±7.61**
Control group	50	72.10±5.34	2.10±0.34	12.65±1.47

### Diagnostic efficacy of GDNF, CEA, and CA199

The sensitivity, specificity, positive predictive value and negative predictive value of the combined detection were 96.0%, 94.0%, 88.9% and 97.9%, see Table 3-Table 4.

**Table 3:** Positive and negative diagnostic results of GDNF, CEA, and CA199.

Pathology	CEA+/-		CA199+/-		GDNF+/-		Combined detection+/-		Total
+	32	18	34	16	38	12	48	2	50
-	10	90	8	92	11	89	6	94	100
**Total **	** 42 **	** 108 **	** 42 **	** 108 **	** 49 **	** 101 **	** 54 **	** 96 **	** 150 **

**Table 4:** Analysis on the diagnostic sensitivity, specificity, positive and negative predictive probability of GDNF, CEA, and CA199.

Groups	Sensitivity (%)	Specificity (%)	Positive predictive value (%)	Negative predictive value (%)
CEA	64.0 (32/50)	90.0 (90/100)	76.2 (32/42)	83.3 (90/108)
CA199	68.0 (34/50)	92.0 (92/100)	81.0 (34/42)	85.2 (92/108)
GDNF	76.0 (38/50)	89.0 (89/100)	77.6 (38/49)	88.1 (89/101)
Combined detection	96.0 (48/50)	94.0 (94/100)	88.9 (48/54)	97.9 (94/96)

### ROC analysis on combined detection of GDNF, CEA, and CA199

**Figure 1:**
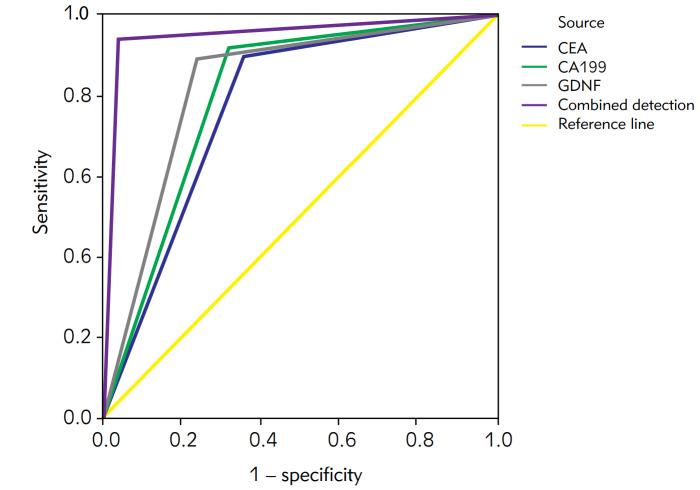
ROC curve of combined detection of GDNF, CEA, and CA199. Notes: In Figure 1, the curve source was CEA, CA199, GDNF, and combined detection, respectively. The yellow line was reference line, the transverse axis was (1-specificity) and the vertical axis was sensitivity.

The combined detection had the AUC (95% CI) = 0.950 (0.909–0.991), the standard error of 0.021, and the progressive Sig. b<0.001, see Figure 1 and Table 5.

**Table 5:** ROC analysis on combined detection of GDNF, CEA, and CA199.

Variables of detection<br>results	AUC	Standard error a	P	Asymptotic 95% CI<br>Upper/lower limits	
CEA	0.770	0.045	<0.001	0.682	0.858
CA199	0.800	0.043	<0.001	0.716	0.884
GDNF	0.825	0.040	<0.001	0.747	0.903
Combined detection	0.950	0.021	<0.001	0.909	0.991

## Discussion

CRC is a malignant tumor in which colorectal glandular epithelial cells develop genetic mutations under the combined action of multiple carcinogens, resulting in unlimited cell proliferation [11]. During the occurrence and development of malignant tumors, tumor cells secrete a variety of tumor markers, while the patients' body also reacts to tumor cells and in turn results in a series of tumor markers [12] [13]. These tumor markers will enter the blood and lymphatic fluid and can be detected in body fluids and excreta, thus reflecting the presence of malignant tumors. One tumor marker can correspond to multiple malignancies, and one malignancy can also correspond to multiple tumor markers, but the diagnostic efficacy of different tumor markers varies in different malignancies [14] [15]. At present, there are several serum tumor markers for the diagnosis of CRC, but their efficacy varies. CEA and CA199 are the most common diagnostic markers for CRC with low sensitivity and specificity, making the combination of multiple serum tumor markers an inevitable choice for the auxiliary diagnosis of CRC at this stage. In this study, patients with CRC and benign colonic diseases were selected as the research subjects. By analyzing the serum levels of GDNF, CEA and CA199 in different groups, the diagnostic efficacy of each single diagnosis and the combined diagnosis was further investigated using ROC curves to provide evidence-based proof for the subsequent diagnosis of CRC. This study found that there was a difference in levels of serum GDNF, CEA and CA199 among the malignant group, benign group and control group, and the diagnostic efficiency of combined detection was higher than that of single detection.

CEA is a soluble protein-polysaccharide complex, which mainly exists on the surface of cancer cells differentiated from endodermal cells and is clinically considered as a specific tumor marker for colorectal adenocarcinoma [16]. On the other hand, the mucin glycoprotein CA199 shows high expression in many gastrointestinal malignancies such as pancreatic cancer, CRC and gastric cancer, which is an important marker for diagnosing gastrointestinal malignancies. Locker GY et al. [17] have concluded that the positive rate of CA199, which is correlated with tumor stages, is 25%-60% in gastric cancer and 18%-58% in CRC. Chen CC et al. [18] believe that CA199 levels rise remarkably in the case of lesion diffusion in gastrointestinal tumors, so CA199 should be used in combination with other markers due to its low specificity when used alone. This study found that CEA and CA199 levels were remarkably higher in the malignant group than in the benign and control groups (P<0.001), confirming that these two markers can reflect tumor changes from different angles. But due to their diagnostic sensitivity of 64.0% and 68.0% respectively, they need to be used in combination with other serum tumor markers.

GDNF is a substance secreted by enteric glial cells, which can nourish enteric neurons under normal physiological conditions and protect them under pathological conditions such as diabetes. The decreased GDNF level indicates a decrease in its function of maintaining the integrity of the intestinal mucosal barrier and the disruption to the homeostasis of the intestinal environment [19] [20] [21]. In this study, the levels of GDNF, which mainly exerts its effects through the GFRαs subunit and the RET receptor tyrosine kinase subunit, were found to be remarkably lower in the malignant group than in the benign and control groups (P<0.001). Receptor tyrosine kinase is an enzyme-linked receptor involved in the proliferation and invasion of a variety of cells, and RET G533C mutation can accelerate the proliferation and invasion of CRC tumor cells. Therefore, RET has a key position in the development and progression of CRC and has been regarded theorized as a new target for its treatment [22] [23] [24]. GDNF can promote the proliferation and metastasis of malignant tumor cells through the RET-SRC-HER2 and RET-AKT signaling pathways. Scholors have found that the GDNF is expressed abnormally in inflammatory bowel disease [25], confirming the specific diagnostic role of GDNF for digestive system diseases. After combining GDNF, the sensitivity of combined detection of GDNF, CEA, and CA199 increased to 96.0%, with specificity as 94.0%, positive predictive value as 88.9%, and negative predictive value as 97.9%. ROC further validated the AUC (95% CI) = 0.950 (0.909-0.991), standard error of 0.021, P<0.001. It is suggested that after combining GDNF, CEA, and CA199 detection, the diagnostic efficacy has risen significantly. Contribution of the study: With the application of ROC curve, the clinical diagnostic value of serum factors selected in this study on CRC can be better understood. This is major clinical progress with broad guiding significance, which can undoubtedly become the direction of future medical development. At the same time, the researchers are supposed to continue to improve the trial design and implement large-sample and multicenter studies in the future, so as to obtain more scientific and accurate evidence-based proof for the benefit of patients.

## Conclusion

To sum up, the combined diagnosis of serum GDNF, CEA and CA199 for CRC has good efficacy, which can improve the detection rate of CRC and is worthy of clinical promotion.

## Dodatak

### Funding

The authors received no financial support for the research, authorship, and publication of this article.

### Data Availability Statement

Data to support the findings of this study is available on reasonable request from the corresponding author.

### Acknowledgements

Not applicable.

### Conflict of interest statement

All the authors declare that they have no conflict of interest in this work.
